# Restoring *HOXD10* Exhibits Therapeutic Potential for Ameliorating Malignant Progression and 5-Fluorouracil Resistance in Colorectal Cancer

**DOI:** 10.3389/fonc.2021.771528

**Published:** 2021-11-01

**Authors:** Weijie Pan, Kaijing Wang, Jiayong Li, Hanhua Li, Yuchan Cai, Min Zhang, Aili Wang, Yazhou Wu, Wei Gao, Wenhao Weng

**Affiliations:** ^1^ Department of Clinical Laboratory, Yangpu Hospital, Tongji University School of Medicine, Shanghai, China; ^2^ Department of Hepatological Surgery, General Surgery, Shanghai East Hospital, Tongji University School of Medicine, Shanghai, China; ^3^ Clinical Laboratory Medicine Center, Shanghai General Hospital Affiliated to Shanghai Jiao Tong University, Shanghai, China; ^4^ Center for Clinical Research and Translational Medicine, Yangpu Hospital, Tongji University School of Medicine, Shanghai, China; ^5^ Institute of Gastrointestinal Surgery and Translational Medicine, Tongji University School of Medicine, Shanghai, China; ^6^ Department of General Surgery, Shanghai General Hospital Affiliated to Shanghai Jiao Tong University, Shanghai, China

**Keywords:** colorectal cancer, *HOXD10*, DNA methylation, miR-7, IGFBP3, 5-fluorouracil, chemosensitivity

## Abstract

Emerging evidence suggests that hypermethylation of *HOXD10* plays an important role in human cancers. However, the biological and clinical impacts of *HOXD10* overmethylation and its downstream targets in colorectal cancer remain unknown. We evaluated the methylation level of *HOXD10* in paired cancer and normal tissues (*n* = 42) by using pyrosequencing, followed by validation of the methylation status of *HOXD10* from The Cancer Genome Atlas (TCGA) datasets with 302 cancer tissues and 38 normal tissues. The biological function of *HOXD10* was characterized in cell lines. We further evaluated the effects of *HOXD10* and its targets on chemoresistance in our established resistant cell lines and clinical cohort (*n* = 66). *HOXD10* was found frequently methylated in colorectal cancer, and its hypermethylation correlates with its low expression level, advanced disease, and lymph node metastasis. Functionally, *HOXD10* acts as a tumor suppressor gene, in which *HOXD10*-expressing cells showed suppressed cell proliferation, colony formation ability, and migration and invasion capacity. Mechanistically, DNMT1, DNMT3B, and MeCP2 were recruited in the *HOXD10* promoter, and demethylation by 5-Aza-2′-deoxycytidine (5-Aza-CdR) treatment or MeCP2 knockdown can sufficiently induce *HOXD10* expression. *HOXD10* regulates the expressions of miR-7 and IGFBP3 in a promoter-dependent manner. Restoration of the expression of *HOXD10* in 5-fluorouracil (5-FU)-resistant cells significantly upregulates the expressions of miR-7 and IGFBP3 and enhances chemosensitivity to 5-FU. In conclusion, we provide novel evidence that *HOXD10* is frequently methylated, silenced, and contributes to the development of colorectal cancers. Restoration of *HOXD10* activates the expressions of miR-7 and IGFBP3 and results in an inhibited phenotype biologically, suggesting its potential therapeutic relevance in colorectal cancer (CRC).

## Introduction

Colorectal cancer (CRC) is the third most common malignant disease worldwide (1, 2). Despite the increasing health burden of CRC, very few breakthroughs have been reported in its treatment. Systemic chemotherapy still remains the mainstay treatment besides surgery and local radiotherapy. 5-Fluorouracil (5-FU)-based chemotherapy, such as FOLFOX and FOLFORI2, is the first-line treatment widely used in advanced CRC (3). However, the prognosis of patients with CRC is often less optimistic than expected, mainly due to 5-FU resistance (4, 5). Several studies have shown that approximately half of patients receiving chemotherapy for stage II and III CRC eventually develop chemotherapy resistance and disease recurrence (6–8). Therefore, identifying potential therapeutical targets has become an imperative need for CRC patients.

Homeobox D10 (*HOXD10*) is a member of the homeobox gene family that serves as a key transcription factor in the development of various types of cancer (9–12). The overexpression of *HOXD10* can reverse miR-23a-mediated invasion in glioblastoma (13). Moreover, *HOXD10* was found to inhibit cell invasion and induce apoptosis *via* the RhoC-AKT signaling pathway (14, 15). Furthermore, suppression of *HOXD10* by miR-10b and miR-1269 was able to promote cancer metastasis in CRC (16, 17). Although these findings showed that *HOXD10* serves as a key regulator in cancer metastasis, the clinical and biological relevance of *HOXD10* in CRC is not fully investigated. In particular, the impact of *HOXD10* on chemosensitivity remains largely unknown in CRC.

DNA methylation, one of the most important epigenetic alterations, plays a crucial role in carcinogenesis (18–20). Accumulating pieces of evidence have shown that frequent hypermethylation of a target gene leads to its reduced expression level, therefore affecting cell behavior (21). Numerous studies have revealed that DNA methylation results in the suppression of *HOXD10* expression in oral squamous cell (22), endometrial (11), and papillary thyroid carcinoma (23). Recently, Yuan et al. (24) observed that *HOXD10* was hypermethylated and low expressed in CRC tissues. However, these results require more clinical data for validation, and the mechanism(s) involved in *HOXD10* methylation needs to be further clarified.

Hence, we systematically and comprehensively investigated the molecular contributions of *HOXD10* methylation in CRC, with a goal to identifying the targets of *HOXD10* to promote colorectal carcinogenesis, finding evidence that indicates the link between *HOXD10* and chemoresistance, and deciphering whether *HOXD10* and its targets may have translational relevance as therapeutic targets. Accordingly, we evaluated the methylation status and the expression of *HOXD10* in CRC tissues from The Cancer Genome Atlas (TCGA) database, Gene Expression Omnibus (GEO) database, and our cohort. We subsequently analyzed the correlation between *HOXD10* methylation and clinical outcomes. Furthermore, we supported these findings by performing a series of functional assays and examining the downstream target genes that contribute to neoplastic progression and chemoresistance in CRC.

## Materials and Methods

### Patients and Specimen Collection

To investigate the methylation status of *HOXD10*, we initially queried TCGA database comprising 302 patients by using the web tool Wanderer, which allows real-time access of the DNA methylation profiles from TCGA (25). To evaluate the prognostic impacts of *HOXD10*, miR-7, and IGFBP3, we analyzed publicly available datasets, including 66 patents who had 5-FU-based chemotherapy from the GSE103479 dataset (26). In the clinical validation cohort, we analyzed 42 frozen tissues, comprising 42 primary CRC tissues, and 42 matched normal mucosa (NM) tissues, which were collected at Shanghai East Hospital, Tongji University School of Medicine. Written informed consent was obtained from all patients, and the study was approved by the Institutional Review Boards of all participating institutions. Patients who had radiotherapy or chemotherapy treatment before surgery were excluded.

### Pyrosequencing Analysis

DNA was extracted from frozen tissues using a QIAamp DNA Mini Kit (Qiagen, Hilden, Germany). Subsequently, 100 ng DNA was bisulfite converted with an Epitect Bisulfite Kit (Qiagen) according to the manufacturer’s protocol. Bisulfite-treated DNA was amplified and then sequenced by pyrosequencing. Biotinylated forward primer: 5′-biotin-GGA GGT TTT TAG AGT TGA GAT TTT-3′; reverse primer: 5′-ACC TTA AAC CCC AAC CTC CTC T-3′. Sequencing primer: 5′-ACA ACA ACC ACA TCTA CT-3′. The methylation levels of the CpG sites were detected and analyzed using PyroMark Q96 ID System (Qiagen).

### Cell Lines

HCT-116 and SW480 were obtained from the American Type Culture Collection (ATCC) and cultured in Dulbecco’s modified Eagle’s medium (DMEM; Invitrogen, Carlsbad, CA, USA) supplemented with 10% fetal bovine serum (FBS) and antibiotics (100 m/ml penicillin and 100 mg/ml streptomycin) at 37°C in 5% humidified CO_2_ atmosphere. The 5-FU-resistant cell lines HCT116/Res and SW480/Res were established in our laboratory and cultured in DMEM with 5 μg/ml of 5-FU.

### Quantitative RT-PCR

Quantitative real-time PCR (qRT-PCR) assays were performed using the ABI7500 Real-Time PCR System (Applied Biosystems, Waltham, MA, USA). For gene expression analysis, the extracted RNA was synthesized to complementary DNA (cDNA) using the RevertAid First Strand cDNA Synthesis Kit (Thermo Scientific, Waltham, MA, USA). The cDNA was then subjected to qPCR using TB Green Premix Ex Taq (Tli RNaseH Plus), ROX plus kit (Takara, Shiga, Japan). The primer sequences for *HOXD10* were as follows: forward: 5′-GAC ATG GGG ACC TAT GGA ATG C-3′, reverse: 5′-CGG ATC TGT CCA ACT GTC TAC T-3′; IGFBP3: forward: 5′-AGA CAC ACT GAA TCA CCT GAA GT-3′, reverse: 5′-AGG GCG ACA CTG CTT TTT CTT-3′; β-actin: forward: 5′-AGA GCT ACG AGC TGC CTG AC-3′, reverse: 5′-AGC ACT GTG TTG GCG TAC AG-3′. The relative expressions of the target genes were normalized against β-actin. For microRNA (miRNA) analysis, qRT-PCR was conducted using the TaqMan MicroRNA Reverse Transcription Kit and TaqMan Universal PCR Master Mix Kit (Applied Biosystems) according to the manufacturer’s instructions. The relative expression of miR-7 was normalized against U6.

### Chromatin Immunoprecipitation Quantitative PCR Assay

Protein–DNA interaction was detected using the SimpleChIP Enzymatic Chromatin IP Kit (Cell Signaling Technology, Danvers, MA, USA). Briefly, the cells were cross-linked using formaldehyde solution, and chromatin was sheared by enzymatic digestion with micrococcal nuclease. The digested chromatin was subsequently incubated with antibodies and protein A/G plus agarose overnight at 4°C. Antibodies, as well as DNMT1, DNMT3B, MeCP2, and *HOXD10*, were purchased from Abcam (Cambridge, UK). The control immunoglobulin G (IgG) was obtained from the Chromatin IP Kit. After immunoprecipitation elution and DNA recovery, the purified DNA was subjected to qPCR. All signals obtained from each immunoprecipitation were expressed as percentages of the total input chromatin.

### Plasmids, siRNA, and miRNA Mimics

*HOXD10*-expressing plasmids, IGFBP3-expressing plasmids, MeCP2 small interfering RNA (siRNA), and miR-7 mimics were all purchased from Shanghai GenePharma Biotech Company (Shanghai, China). CRC cells were transfected with plasmids, siRNA, or mimics using Lipofectamine 3000 (Invitrogen) and Opti-MEM (Gibco, Grand Island, NY, USA) according to the manufacturers’ instructions.

### Cell Viability and Colony Formation Assay

The cell viability of CRC with different treatments was detected using Cell Counting Kit-8 (CCK-8, Beyotime Institute of Biotechnology, Haimen, China) according to the manufacturer’s instructions. Briefly, the cells with different treatments were cultured for 1–5 days and then 10 µl of CCK-8 reagent was added to each well. After 2 h of incubation, the absorbance was measured at 450 nm using a microplate reader. For the colony formation assay, 500 cells were seeded in each well of six-well plates and incubated for 10 days. The colonies were stained with crystal violet and counted.

### Migration and Invasion Assays

Boyden chambers (Corning, Corning, NY, USA) with 8-mm pore size membranes coated with Matrigel (for invasion assays) or without Matrigel (for migration assays) were used for the cell migration and invasion assays. *HOXD10*-expressing cells or control cells were seeded onto the inserts at 5 × 10^5^ cells in serum-free medium and transferred into wells with culture medium containing 10% FBS. After 24 h incubation, the non-invading cells on the top surface of the membrane were removed, while the invaded cells on the bottom of the membrane were fixed and stained using the Diff-Quik Staining Kit (Thermo Scientific) according to the manufacturer’s instructions. The stained cells were therefore counted using a light microscope.

### Luciferase Reporter Assays

DNA fragments with *HOXD10* binding sequences or deleted mutant sequences were ligated into the pGL4.21 firefly luciferase reporter vectors (Promega, Madison, WI, USA) according to the manufacturer’s instructions. HCT116 and SW480 cells were co-transfected with pGL4.21 reporter vectors and a Renilla luciferase plasmid, incubated for 48 h, and harvested according to the manufacturer’s instructions. Luciferase activities were measured using a dual-luciferase reagent (Promega). The luciferase activity was quantified by normalizing the signal of firefly to the activity of Renilla.

### Statistical Analysis

All statistical analyses were performed using GraphPad Prism version 6.0 or MedCalc version 12.3 programs. Statistical differences between groups were determined with independent *t*-test or the Mann–Whitney *U* test. Median values were used as the cutoff values to determine the high expression or the low expression of *HOXD10*. Kaplan–Meier analysis and log-rank test were used to estimate and compare the progression-free survival (PFS) rates of CRC patients with high and low *HOXD10* expressions. All *p*-values were two-sided, and those less than 0.05 were considered statistically significant.

## Results

### *HOXD10* Is Frequently Methylated in Colorectal Cancer and Its Hypermethylation Correlates with Advanced Disease and Metastasis

**Figure 1:**
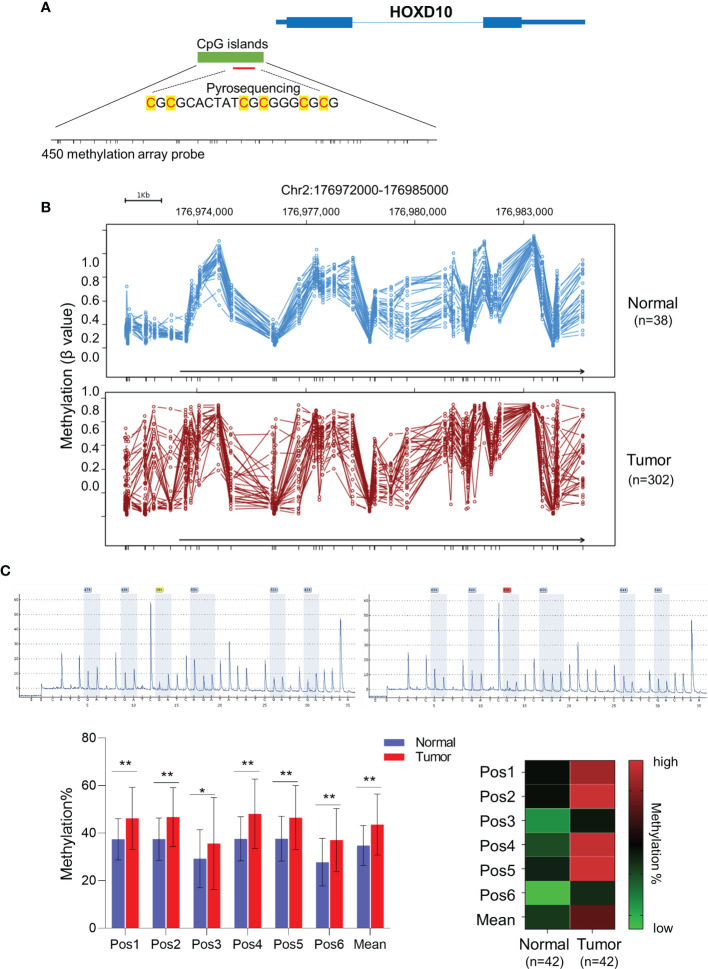
*HOXD10* is frequently methylated in colorectal cancer. **(A)** Illustration of the CpG sites for pyrosequencing and HumanMethylation450 BeadChip loci in the promoter region of *HOXD10*. **(B)** The web tool Wanderer showed the methylation value of each 450 methylation loci in normal and colorectal cancer tissues. **(C)** Representative images of the pyrosequencing data are shown on the *top left* (#1 normal tissue) and *top right* (#1 cancer tissue). The *bottom left* image shows the methylation value of six CpG sites from 42 cancer tissues and normal tissues. *p < 0.05, **p < 0.01 (Mann–Whitney test). The *bottom right image* shows the heatmap of the methylation value from the six CpG sites.

To investigate the role of *HOXD10* methylation in CRC, we first evaluated *HOXD10* methylation in tumor and normal tissues from TCGA database and our cohort. **Figure 1A** shows HumanMethylation450 BeadChip loci in the vicinity of the *HOXD10* promoter. By using the web tool Wanderer, we obtained the methylation profiles of *HOXD10* from TCGA (**Figure 1B**). Several CpG sites of *HOXD10* were observed frequently hypermethylated in tumor tissues (*n* = 302) compared to that in normal tissues (*n* = 38) (**Figure 1B** and **Supplementary Table S1**). Subsequently, we performed pyrosequencing analysis to measure the methylation level of *HOXD10* in our cohort. We designed six CpG sites in our pyrosequencing analysis (**Figure 1A**), and our results showed that the average methylation levels of these six CpG site were higher in tumor tissues than those in normal tissues [43.03% (35.21%–53.27%) *vs.* 28.34% (22.03%–35.20%), *n* = 42, *p* < 0.0001] (**Figure 1C**).

**Figure 2:**
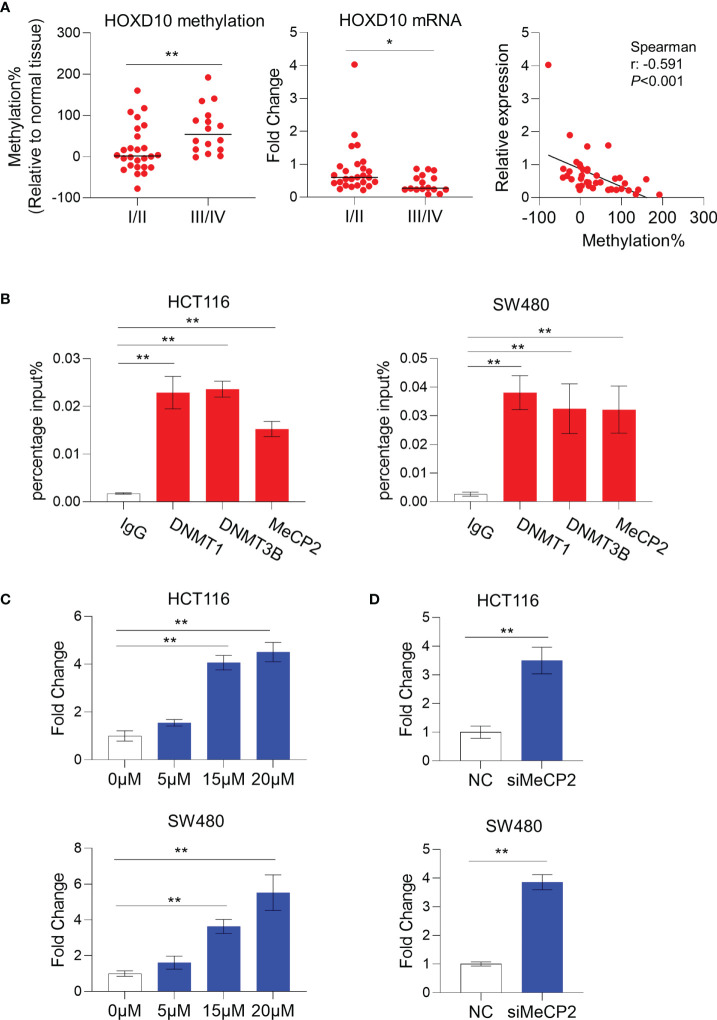
*HOXD10* methylation correlated with its low expression in colorectal cancer. **(A)** The relative methylation and mRNA levels of *HOXD10* were demonstrated. Patients with advanced stage (III/IV) cancer had a higher level of methylation, but a lower expression level, compared to those with early stage (I/II) cancer. *p < 0.05, **p < 0.01 (Mann–Whitney test). Correlation analysis was also performed and showed that *HOXD10* methylation was negatively associated with its expression level. **(B)** Chromatin immunoprecipitation quantitative PCR (ChIP-qPCR) assay revealed a significant enrichment of DNMT1, DNMT3B, and MeCP2 observed in the *HOXD10* promoter region in the colorectal cancer cell lines HCT116 and SW480. **p < 0.01 (independent *t*-test). **(C)** HCT116 and SW480 cells were treated with the DNA methyltransferase (DNMT) inhibitor 5-Aza-2′-deoxycytidine (5-Aza-CdR) at concentrations ranging from 0 to 20 μM. The expression of *HOXD10* was then evaluated by qPCR. **p < 0.01 (ANOVA test). **(D)** The expression of *HOXD10* was examined in *HOXD10*-expressing cells and control cells using qPCR. **p < 0.01 (independent *t*-test).

We next evaluated the expression pattern of *HOXD10* methylation in the context of its clinical significance in our cohort. *HOXD10* hypermethylation was observed in a stage-dependent manner (*p* = 0.0120) (**Table 1** and **Figure 2A**). Furthermore, *HOXD10* hypermethylation was significantly more pronounced in cancer tissues with lymph node metastasis (*p* = 0.0120) (**Table 1**), suggesting that *HOXD10* hypermethylation plays a key role in the development of CRC.

**Table 1:** Correlation between *HOXD10* methylation and clinicopathologic features.

Variables		*HOXD10* methylation		*p*-value
		Low (*n* = 21)	High (*n* = 21)	
Age (years)	≤54	12	11	0.1610
	>54	9	10	
Gender	Female	6	9	0.340
	Male	15	12	
Tumor site	Rectum	5	4	0.710
	Colon	16	17	
Differentiation	Well/moderate	18	17	0.683
	Poor	3	4	
Tumor size (cm)	<3.5	9	9	1.000
	≥3.5	12	12	
Venous invasion	Negative	18	15	0.265
	Positive	3	6	
Nerve invasion	Negative	15	13	0.518
	Positive	6	8	
T stage	T1/T2	6	4	0.474
	T3/T4	15	17	
Lymph node metastasis	Negative	17	9	0.0120**
	Positive	4	12	
Liver metastasis	Negative	20	18	0.2999
	Positive	1	3	
TNM	I/II	17	9	0.0120**
	III/IV	4	12	

**p < 0.01.

### DNA Methylation Leads to the Downregulation of *HOXD10* Expression in Colorectal Cancer

To investigate the correlation between *HOXD10* methylation and its expression, we measured the messenger RNA (mRNA) level of *HOXD10* in our cohort. We found that *HOXD10* expression was decreased, while its methylation level increased, in a stage-dependent manner (**Figure 2A**). Furthermore, *HOXD10* expression showed a significant negative correlation with its methylation (*r* = −0.591, *p* < 0.001) (**Figure 2A**), suggesting that *HOXD10* hypermethylation may contribute to its downregulation in CRC.

Accumulating evidence has demonstrated that DNA methyltransferases (DNMTs) frequently methylate the cytosine within CpG dinucleotides, and the methylated CpGs recruit a plethora of interacting proteins, such as methyl-CpG binding protein (MeCPs) and methyl-CpG binding domains (MBDs), to repress transcription (27, 28). By using chromatin immunoprecipitation (ChIP) assay, a significant enrichment of DNMT1, DNMT3B, and MeCP2 was observed in the *HOXD10* promoter region in the CRC cell lines HCT116 and SW480 (**Figure 2B**). Furthermore, we treated CRC cells with the DNMT inhibitor 5-Aza-2′-deoxycytidine (5-Aza-CdR) and subsequently measured the expression and methylation levels of *HOXD10*. As shown in **Figure 2C**, 5-Aza-CdR treatment strikingly induced the expression of *HOXD10*, with an approximately fivefold increase in 5-Aza-CdR-treated cells compared to that in control cells (*p* < 0.01). Likewise, we have successfully reduced the expression of MeCP2 by using specific siRNA in HCT116 and SW480 cells, and the expression of *HOXD10* was remarkably increased (*p* < 0.01) (**Figure 2D**). These findings highly support our hypothesis that DNA methylation caused the decreased *HOXD10* expression in CRC.

### Restoring *HOXD10* Expression Suppresses Cell Growth and Invasiveness in Colorectal Cancer Cells

**Figure 3:**
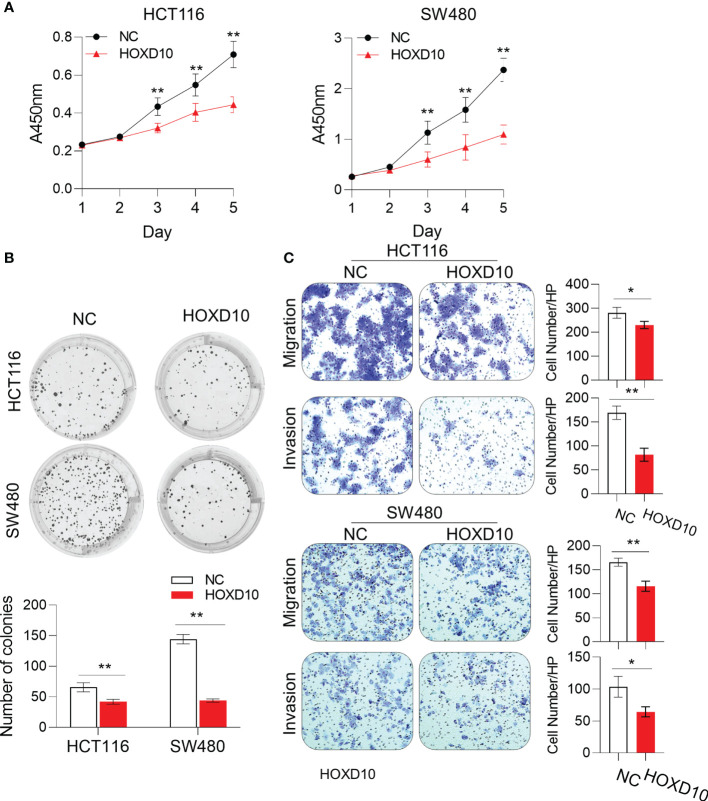
*HOXD10* inhibited cell growth, colony formation, and migration and invasion. HCT116 and SW480 cells were transfected with either *HOXD10* overexpression plasmids or control empty plasmids. *HOXD10*-expressing cells and control cells were then used for the CCK-8 assay **(A)**, colony formation assay **(B)**, and migration and invasion assays **(C)**. *p < 0.05, **p < 0.01 (independent *t*-test comparing control and treated cells).

To investigate whether the restoration of *HOXD10* could affect the biological functions in CRC cells, we generated the *HOXD10*-expressing HCT116 and SW480 cells. The CCK-8 assay showed that ectopic expression of *HOXD10* significantly inhibited cell proliferation in both HCT116 and SW480 cell lines (**Figure 3A**). Moreover, colony formation was significantly inhibited in *HOXD10*-overexpressing cells compared to that in control cells (**Figure 3B**). To determine whether the overexpression of *HOXD10* has any impact on the motility and invasive potential of CRC cells, we performed migration and invasion assays. *HOXD10*-expressing cells showed a remarkably impaired migration and invasion ability in both cancer cell lines compared with that in control cells (**Figure 3C**). Together, these results suggest that *HOXD10* exerts tumor-suppressive functions in CRC.

### *HOXD10* Regulates miR-7 and IGFBP3 Expressions in Colorectal Cancer

**Figure 4:**
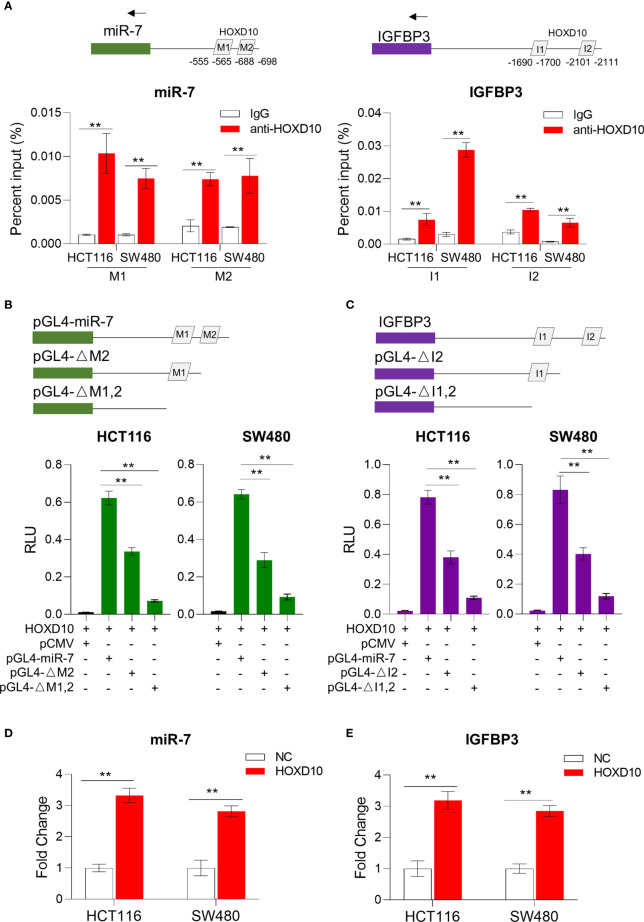
*HOXD10* regulated miR-7 and IGFBP3 in a promoter-dependent manner. **(A)** Chromatin immunoprecipitation quantitative PCR (ChIP-qPCR) assay revealed that *HOXD10* binds to the miR-7 and IGFBP3 promoter region in HCT116 and SW480 cells. **p < 0.01 (independent *t*-test comparing IgG control and *HOXD10* pull-down). **(B, C)** Luciferase reporter assay demonstrated the effect of transient expression of *HOXD10* on either wild or deleted mutant promoter of miR-7 and IGFBP3. **p < 0.01 (independent *t*-test). **(D, E)** Relative expressions of miR-7 and IGFBP3 in *HOXD10*-expressing cells and control cells by qPCR. **p < 0.01 (independent *t*-test comparing control and *HOXD10*-expressing cells).

Previous studies have demonstrated miR-7 and IGFBP3 as direct targets of *HOXD10* (29–32); however, whether *HOXD10* functions through miR-7 or IGFBP3 in CRC remains unknown. By using the online web tool PROMO (33), a virtual laboratory for the identification of putative transcription factor binding sites (TFBS) in DNA sequences, we predicated the potential binding sites of *HOXD10* in the miR-7 and IGFBP3 promoter region (**Figure 4A**) and subsequently validated it using ChIP assay. Consistently, we observed a strong binding signal of *HOXD10* in the miR-7 and IGFBP3 promoter region in HCT116 and SW480 cells (**Figure 4A**).

We further cloned the putative miR-7 and IGFBP3 promoters into a TATA-less basic pGL4-luc reporter (pGLmiR-7) and also constructed a mutant reporter with a series of deleted *HOXD10* binding sequences. As shown in **Figures 4B, C**, the transient expression of *HOXD10* significantly stimulated the transcription of miR-7 and IGFBP3 from the wild luciferase reporter, but showed less effect on the expressions of those from the deleted mutant. Moreover, the overexpression of *HOXD10* led to an obvious upregulation of miR-7 and IGFBP3 in CRC cell lines (**Figures 4D, E**).

**Figure 5:**
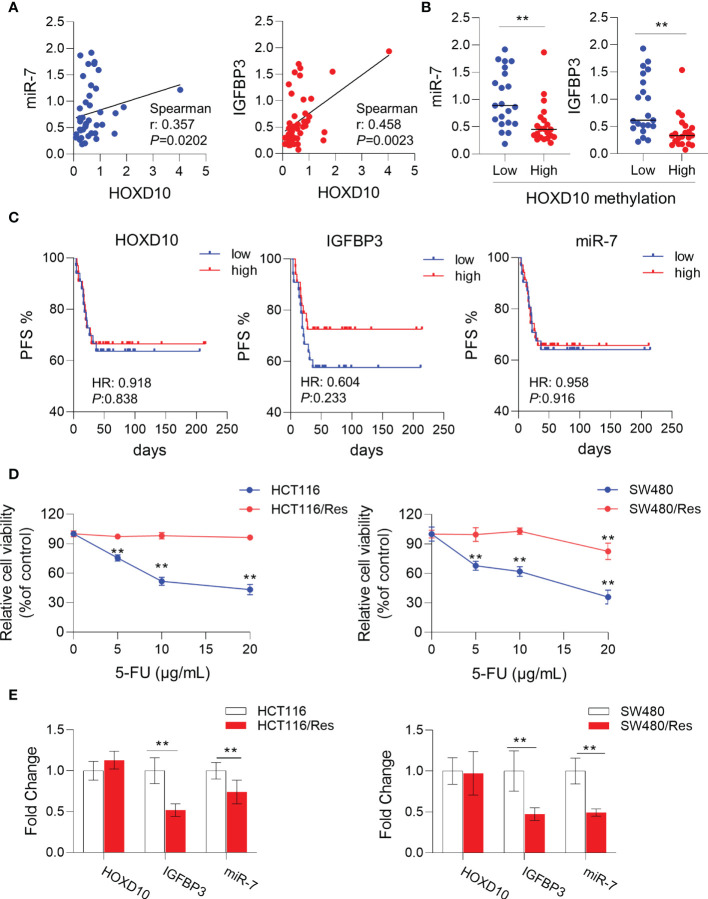
*HOXD10* and its targets miR-7 and IGFBP3 were correlated with 5-fluorouracil (5-FU)-resistant in colorectal cancer. **(A)** Correlation of the expressions of *HOXD10* and its targets, miR-7 and IGFBP3. **(B)** Correlation of *HOXD10* methylation and the expression of either miR-7 or IGFBP3. **p < 0.01 (Mann–Whitney test). **(C)** Correlation of *HOXD10*, miR-7, and IGFBP3 with progression-free survival in stage II and III CRC patients (*n* = 66) treated with adjuvant 5-FU-based chemotherapy using a publicly available GEO dataset (GSE103479). **(D)** CCK-8 assay showed the cell viability of 5-FU-resistant cell lines, HCT116/Res and SW480/Res, and their parental cells in the presence of 5-FU at different concentrations. **p < 0.01 (independent *t*-test comparing parental and 5-FU-resistant cells). **(E)** Relative expressions of *HOXD10*, miR-7, and IGFBP3 in parental and 5-FU-resistant cells by qPCR. **p < 0.01 (independent *t*-test comparing parental and 5-FU-resistant cells).

To further validate our *in vitro* results that *HOXD10* regulated miR-7 and IGFBP3, we investigated the correlation of the expressions between *HOXD10* and its target genes in CRC tissues. As shown in **Figure 5A**, the expression of *HOXD10* was positively associated with the expressions of miR-7 (*r* = 0.357, *p* < 0.05) and IGFBP3 (*r* = 0.458, *p* < 0.01). Furthermore, cancer tissues with methylated *HOXD10* revealed low expressions of both miR-7 and IGFBP3 (*p* < 0.01) (**Figure 5B**). Together, these observations suggest that *HOXD10* directly interacts with the putative miR-7 and IGFBP3 promoters and that the expressions of these genes are positively regulated by the transcription factor *HOXD10*.

### Restoration of *HOXD10* Expression Serves as a Potential Therapeutic Target in Colorectal Cancer

Previous studies have demonstrated that miR-7 (34–37) and IGFBP3 (38–40) had significant impacts on chemosensitivity in a variety of cancers. We therefore assumed that *HOXD10* and its targets, miR-7 and IGFBP3, may have clinical relevance as therapeutic targets for enhancing 5-FU sensitivity in CRC. To this end, we investigated the correlation of the expressions of *HOXD10* and its targets with PFS in stage II and III CRC patients (*n* = 66) treated with adjuvant 5-FU-based chemotherapy using a publicly available GEO dataset (GSE103479). Although *HOXD10* and its targets failed to show a significant correlation with PFS, we indeed observed a trend that patients with low expressions of *HOXD10*, miR-7, and IGFBP3 had poor prognosis (**Figure 5C**), suggesting that the activation of *HOXD10* and its targets may increase chemosensitivity to 5-FU in CRC.

**Figure 6:**
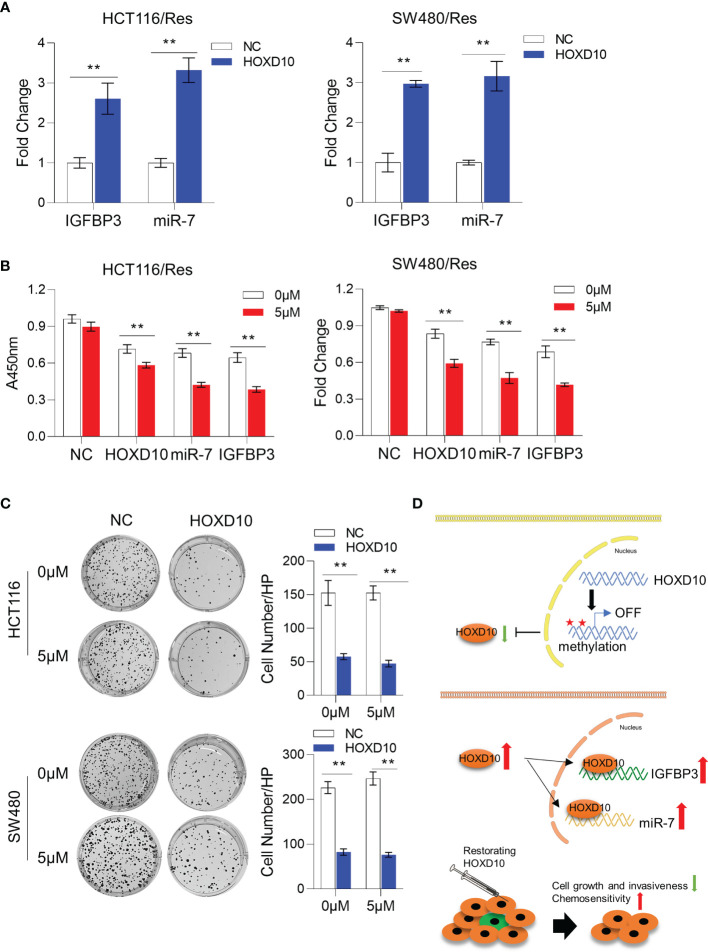
Restoration of *HOXD10* in resistant cells enhanced chemosensitivity through miR-7 and IGFBP3. **(A)** Relative expressions of miR-7 and IGFBP3 in resistant colorectal cancer cells with *HOXD10* overexpression or control cells. **p < 0.01 (independent *t*-test comparing control and *HOXD10*-expressing cells). **(B)** CCK-8 assay showed the cell viability of resistant cells with *HOXD10*, miR-7, or IGFBP3 overexpression under treatment with 5 μg/ml 5-fluorouracil (5-FU). **p < 0.01 (independent *t*-test). **(C)** Colony formation assay showed that resistant cells expressing *HOXD10* showed a significantly reduced number of colonies compared to control cells in either absence or presence of 5-FU. **p < 0.01 (independent *t*-test). **(D)** Schematic illustration showing that *HOXD10* is frequently methylated, silenced, and contributes to the development of colorectal cancers. The re-expressed *HOXD2* directly regulates the expressions of both miR-7 and IGFBP3, highlighting that the restoration of *HOXD10* with concurrent 5-FU-based chemotherapy may be a potential treatment option for patients with CRC.

We have successfully established 5-FU-resistant cell lines, HCT116/Res and SW480/Res. As shown in **Figure 5D**, the resistant cell lines can grow in culture medium supplemented with 5–20 μg/ml 5-FU, while the parental cell lines showed inhibited cell growth in the presence of 5 μg/ml of 5-FU. We then measured the expression levels of *HOXD10*, miR-7, and IGFBP3 in parental and resistant cell lines. Interestingly, miR-7 and IGFBP3 were significantly downregulated in resistant cell lines compared to that in parental cells (**Figure 5E**), while the expression of *HOXD10* was not obviously altered in resistant cells, suggesting that *HOXD10* was possibly methylated in both parental and resistant cells. Therefore, we hypothesized that restoring the expression of *HOXD10* in resistant cells may activate the expressions of miR-7 and IGFBP3. In line with our assumption, the overexpression of *HOXD10* strikingly upregulated miR-7 and IGFBP3 in the resistant cell line (**Figure 6A**).

To further determine whether restoring *HOXD10* facilitates chemosensitization *via* miR-7 and IGFBP3, we compared the cell viability of control resistant cells and resistant cells expressing *HOXD10*, IGFBP3, or miR-7 in the presence of 5 μg/ml of 5-FU. Consistent with previous data, 5-FU treatment did not have any growth inhibitory effect on controls cells, while *HOXD10*, IGFBP3, or miR-7 significantly suppressed cell growth under treatment of 5-FU, suggesting that *HOXD10* increases 5-FU sensitivity through miR-7 and IGFBP3 (**Figure 6B**). We further validated these results by performing colony formation assay. We observed that resistant cells expressing *HOXD10* showed a significantly reduced number of colonies compared to control cells in either absence or presence of 5-FU (**Figure 6C**). Collectively, our analyses provided evidence that restoring the expression of *HOXD10* can sufficiently increase the expressions of miR-7 and IGFBP3, therefore inhibiting 5-FU resistance in CRC (**Figure 6D**).

## Discussion

CRC is one of the most common malignant diseases worldwide. Therefore, elucidation of the molecular mechanisms involved in the development of CRC is essential for the identification of novel biomarkers or better therapeutic targets for the management of CRC patients. Numerous studies have shown that adjuvant 5-FU-based chemotherapy can improve the 5-year overall survival (OS) of stage II and III patients compared to surgery alone (41, 42). However, the prognosis of such patients still remains poor, mainly due to 5-FU resistance (43, 44). Herein, we have made several novel observations. Firstly, we found that *HOXD10* was frequently methylated in CRC and that overmethylated *HOXD10* was associated with tumor stage and lymph node metastasis. Secondly, we revealed the mechanism that a plethora of methylation-associated proteins were recruited to the promoter of *HOXD10*, therefore leading to the inhibition of transcription. *HOXD10* methylation was negatively correlated with its expression. Thirdly, we showed the biological relevance of *HOXD10* as a tumor-suppressive DNA in CRC. Fourthly, we demonstrated that *HOXD10* positively regulated miR-7 and IGFBP3 *in vitro*. Restoration of *HOXD10* can suppress CRC cell growth and invasiveness, as well as 5-FU resistance, highlighting its potential therapeutic role in the management of patients with CRC.

In view of recent limited evidence that *HOXD10* methylation contributes to gene regulation and carcinogenesis, in the present study, we first revealed that *HOXD10* was differentially methylated between cancer and normal tissues, which was observed from both TCGA dataset and our cohort. We further demonstrated that *HOXD10* methylation correlated with advanced stage and lymph node metastasis, suggesting that epigenetically silenced *HOXD10* contributes to the progress of CRC. Interestingly, *HOXD10* methylation occurs not only in the CRC but also in other types of cancer, such as oral squamous cell carcinoma (22), endometrial carcinoma (11), and hepatocellular carcinoma (45), suggesting that *HOXD10* hypermethylation is a frequent driver event and represents a promising pan-cancer therapeutic target.

We have previously reported that miR-7 activity can be regulated by circular RNA ciRS-7 in CRC (46). The ciRS-7-inhibited activity led to the impaired oncogenic phenotype of CRC cells. Herein, we discovered a new mechanism that *HOXD10* stimulates miR-7 expression in a promoter-dependent manner. We confirmed the binding sequence of *HOXD10* in the miR-7 promoter region. The overexpression of *HOXD10* can sufficiently upregulate the promoter activity and transcription level of miR-7, suggesting that *HOXD10* exerts its tumor-suppressive function, at least in part, through miR-7. Recently, miR-7 was found to contribute to chemoresistance in multiple cancers through its targets, such as YAP, MRP1, BCL2, YY1, PARP1 (34, 35, 47, 48). However, the impact of miR-7 on chemoresistance in CRC remains largely unknown. Unfortunately, we cannot find clinical evidence that miR-7 can affect the prognosis of CRC patients who had 5-FU treatment, possibly due to the clinically and biologically heterogeneous malignancy of this disease or the lack of sufficient number of participants in our investigated cohort. Our *in vitro* data, however, showed that 5-FU-resistant cells showed lower expression levels of miR-7 compared to those in parental CRC cells. Furthermore, the overexpression of miR-7 in resistant cells increased their sensitivity to 5-FU, implicating that miR-7 substantially increases 5-FU sensitivity in CRC.

Likewise, IGFBP3 is another direct target of *HOXD10* in CRC. We found that the ectopic expression of *HOXD10* significantly induced IGFBP3 promoter activity and its expression in CRC. A few studies have suggested the downregulation of IGFBP3 in CRC and its role in the regulation of invasion and metastasis (49–51). Consistent with this, our study observed that *HOXD10* suppressed cell migration and invasion and that the low level of *HOXD10* was associated with metastasis in CRC, highlighting the critical role of IGFBP3 in *HOXD10*-mediated tumor-suppressive effect. One study, however, indicated that IGFBP-3 showed biological effects on the chemosensitization of pancreatic ductal adenocarcinoma cells (52). Until now, studies reporting the functional or clinical significance of IGFBP3 in association with chemoresistance in CRC are limited. Interestingly, we observed a trend that 5-FU-treated patients with low expressions of IGFBP3 had poor PFS, supporting our hypothesis that IGFBP3 was involved in chemoresistance. Our *in vitro* data validated IGFBP3 being able to increase 5-FU sensitivity in resistant cells. These findings may help provide a better understanding of the mechanisms of *HOXD10* and its interacting targets, miR-7 and IGFBP3, in cancer progression and chemoresistance in CRC, suggesting that *HOXD10* may serve as an important potential therapeutic target in this disease.

To fully appreciate the therapeutic relevance of *HOXD10* in CRC, its biological significance as an inhibitor to colorectal pathogenesis should also be considered. Our functional experiments provided convincing evidence to support the correlation of *HOXD10* with an inhibited phenotype, in which *HOXD10* overexpression suppressed cell proliferation, colony formation ability, and the migration and invasion capacity. Moreover, the restoration of *HOXD10* in 5-FU-resistant cells efficiently induced the expressions of miR-7 and IGFBP3, therefore sensitizing resistant cells to 5-FU. Accordingly, our results successfully proved our hypothesis, whereby *HOXD10* activated the expressions of miR-7 and IGFBP3 and resulted in a biologically inhibited phenotype. However, we did not find an obvious effect of *HOXD10* on the prognosis of patients who had 5-FU treatment. Additional cohort studies with large patient numbers are required.

Taken together, we provide novel evidence that *HOXD10* is frequently methylated, silenced, and contributes to the development of CRC. From a functional and mechanism perspective, *HOXD2* directly regulates the expression of both miR-7 and IGFBP3. We conclude that the restoration of *HOXD10* with concurrent 5-FU-based chemotherapy may be a potential treatment option for patients with this malignant disease.

## Data Availability Statement

The original contributions presented in the study are included in the article/**Supplementary Material**. Further inquiries can be directed to the corresponding authors.

## Ethics Statement

The studies involving human participants were reviewed and approved by Shanghai East Hospital, Tongji University School of Medicine. The patients/participants provided written informed consent to participate in this study.

## Author Contributions

WP, YW, WG, and WW contributed to the study concept and design. KW and WG provided the specimen. KW acquired clinical data. WP, JL, HL, YC, MZ, AW, and YW performed the *in vitro* experiment. WP and WW drafted the manuscript. All authors contributed to the article and approved the submitted version.

## Funding

The present work was supported by grants no. 81672826 and no. 81874179 from the National Natural Science Foundation of China, no. 2017YQ044 from the Municipal Human Resources Development Program for Outstanding Young Talents in Medical and Health Sciences in Shanghai, 18PJD047 from Shanghai Pujiang Talent Plan, and no. 18411969900 from the Science and Technology Commission of Shanghai Municipality to WW. The views expressed in the submitted article are the authors’ own and not an official position of the institutions or funders.

## Conflict of Interest

The authors declare that the research was conducted in the absence of any commercial or financial relationships that could be construed as a potential conflict of interest.

## Publisher’s Note

All claims expressed in this article are solely those of the authors and do not necessarily represent those of their affiliated organizations, or those of the publisher, the editors and the reviewers. Any product that may be evaluated in this article, or claim that may be made by its manufacturer, is not guaranteed or endorsed by the publisher.
